# Mapping and genotypic analysis of the *NK-lysin* gene in chicken

**DOI:** 10.1186/1297-9686-46-43

**Published:** 2014-07-07

**Authors:** Mi Ok Lee, Ence Yang, Mireille Morisson, Alain Vignal, Yong-Zhen Huang, Hans H Cheng, William M Muir, Susan J Lamont, Hyun Soon Lillehoj, Sung Hyen Lee, James E Womack

**Affiliations:** 1 Department of Veterinary Pathobiology, Texas A & M University, College Station, TX 77843, USA; 2 Department of Veterinary Integrative Biosciences, Texas A & M University, College Station, TX 77843, USA; 3 INRA, UMR1388 Génétique, Physiologie et Systèmes d’Elevage, Castanet-Tolosan F-31326, France; 4 Université de Toulouse INPT ENSAT, UMR1388 Génétique, Physiologie et Systèmes d’Elevage, Castanet-Tolosan F-31326, France; 5 Université de Toulouse INPT ENVT, UMR1388 Génétique, Physiologie et Systèmes d’Elevage, Toulouse F-31076, France; 6 College of Animal Science and Technology, Northwest A&F University, Yangling, Shaanxi, People’s Republic of China; 7 USDA, ARS, Avian Disease and Oncology Laboratory, East Lansing, MI 48823, USA; 8 Department of Animal Science, Purdue University, West Lafayette, IN 47907, USA; 9 Department of Animal Science, Iowa State University, Ames, IA 50011, USA; 10 USDA, ARS, Animal Biosciences and Biotechnology Laboratory, Beltsville Agricultural Research Center, Beltsville, MD 20705, USA

## Abstract

**Background:**

Antimicrobial peptides (AMP) are important elements of the first line of defence against pathogens in animals. NK-lysin is a cationic AMP that plays a critical role in innate immunity. The chicken *NK-lysin* gene has been cloned and its antimicrobial and anticancer activity has been described but its location in the chicken genome remains unknown. Here, we mapped the *NK-lysin* gene and examined the distribution of a functionally significant single nucleotide polymorphism (SNP) among different chicken inbred lines and heritage breeds.

**Results:**

A 6000 rad radiation hybrid panel (ChickRH6) was used to map the *NK-lysin* gene to the distal end of chromosome 22. Two additional genes, the *adipocyte enhancer-binding protein 1-lik*e gene (*AEBP1*) and the *DNA polymerase delta subunit 2-like* (*POLD2*) gene, are located in the same NW_003779909 contig as *NK-lysin*, and were thus indirectly mapped to chromosome 22 as well. Previously, we reported a functionally significant SNP at position 271 of the *NK-lysin* coding sequence in two different chicken breeds. Here, we examined this SNP and found that the A allele appears to be more common than the G allele in these heritage breeds and inbred lines.

**Conclusions:**

The chicken *NK-lysin* gene mapped to the distal end of chromosome 22. Two additional genes, *AEBP1* and *POLD2*, were indirectly mapped to chromosome 22 also. SNP analyses revealed that the A allele, which encodes a peptide with a higher antimicrobial activity, is more common than the G allele in our tested inbred lines and heritage breeds.

## Background

The chicken is an important animal for several reasons. In addition to being a major source of protein in the world, it is valuable to the understanding of genome evolution because of its relationship to mammals. The chicken genome sequence assembly was completed in 2004 with a six-fold whole genome shotgun (Sanger) coverage. It was the first avian genome to be sequenced [1] and, therefore, holds a place in comparative genomics as a prototypic avian genome. Subsequent sequencing and mapping have improved upon that first build, and efforts continue in search of missing and/or unassembled sequences, primarily on the smaller microchromosomes and the sex chromosomes. Nevertheless, sequence segments remain misplaced in the genome assembly [2] or unmapped.

Radiation hybrid (RH) panels are useful mapping tools to determine the location and order of genes, and to aid the assembly of genome sequences. RH panels are available for several domestic animal species including cow [3], pig [4], horse [5], dog [6], cat [7], mouse [8], chicken [9] and duck [10]. The ChickRH6 radiation hybrid panel was produced in 2002 [9] and has been used to construct consensus chromosome RH maps of the chicken genome and a limited number of chicken or GGA (*Gallus gallus*) chromosome maps have been published to date, namely GGA2 [11], GGA4 [12], GGA5 [13], GGA7 [14], GGA14 [15], GGA15 [16], GGA16 [17] and GGA25 [18].

NK-lysin is a cationic peptide with antibacterial activity that was originally isolated from porcine intestinal tissue. Extensive research has been conducted on the structure and antimicrobial activities of NK-lysin isolated from different species [19-23]. The chicken *NK-lysin* gene was cloned in 2006 [24], and its antimicrobial activity against *Eimeria sporozocites* was reported [25]. Previously, we identified a single nucleotide polymorphism (SNP) in the chicken *NK-lysin* gene and discovered that the encoded protein variants are differentially cytotoxic for several bacteria and cell lines derived from human cancers [26]. However, to date the location of *NK-lysin* on the chicken genome remains unknown. Here, we used the ChickRH6 panel to map the *NK-lysin* gene on the chicken genome, and also examined its polymorphism and allele distribution among diverse heritage breeds and inbred lines of chicken.

## Methods

### Genotyping

Different heritage chicken breeds available publically and several experimental inbred lines were surveyed. The heritage chicken breed samples came as pooled blood from eight to 26 animals, each. Three to seven individual samples were used for each inbred line. DNA (50 ng) was used for PCR amplification with a forward primer of cNKL QF3 and reverse primer of cNKL QR2 (Table 1), and Sanger sequencing was carried out with the BigDye Termination kit, version 1 (ABI, Foster City, CA). To estimate allele frequencies from pooled blood samples, mixtures of known amounts of pure A and G allele DNA at ratios of 1:0, 3:1, 2:1, 1:1, 1:2 and 0:1 were prepared as a standard reference for quantitative PCR products. The peak area and heights were measured to approximate the ratio of alleles in pooled DNA from the heritage breed samples.

**Table 1:** Primer sequences used

**Primer pairs**	**5′= > 3′**	**Size (bp)**	**Tm (°C)**	**Reference for primers GenBank accession number**
cNKL QF3	GATGCAGATGAAGGGGACGC	278	62	NM_001044680
cNKL QR2	CTGCCGGAGCTTCTTCAACA			
NUDCD3 F	TCCTCTCTCCAAGTGCGTTT	248	62	XM_004947649
NUDCD3 R	TACACCTACACTCGCCAGCA			
ADRA1A F	CTGTAGCCGACCTCCTCTTG	182	62	XM_425762
ADRA1A R	GCTCACCCCGATGTATCTGT			
LRRTM4 F	GTTCTGCAGGAGTGGGGTTA	165	62	XM_004947641
LRRTM4 R	GGTAATGGGAGGCAACAAGA			
SLC20A F	CAAAGTCAGCGAGACCAT CC	218	62	XM_003642557
SLC20A R	ATGGGAAGCTTCAAGAACGA			

### Radiation hybrid panel genotyping and map construction

The chicken *NK-lysin* gene was physically mapped using the INRA Chicken RH panel (ChickRH6) containing 90 hamster-chicken hybrid cell lines [9]. DNA from each line, along with control chicken and hamster DNA, were analysed for the presence or absence of *NK-lysin* and other markers by PCR (polymerase chain reaction) in 96-well microtiter plates. PCR was performed with the primers listed in Table 1. The PCR reactions were conducted with an initial denaturation step of 95°C for 5 min, followed by 35 cycles of 95°C for 30 s, 62°C for 30 s, 72°C for 30 s, and a final elongation step of 5 min at 72°C. Each marker was run at least twice on the RH panel to insure reproducibility. The amplified product was typed and scored as present (1), absent (0), or ambiguous (2) as previously described [9].

Chromosome assignment was done and mapping was performed by including our genotyping data in a larger dataset composed of 10 143 markers for the chicken genome. This dataset is composed of 2663 markers (genes and microsatellites) from the ChickRH database (http://chickrh.toulouse.inra.fr) and 7480 SNPs genotyped on the ChickRH panel using the Illumina GoldenGate assay at the Centre National de Génotypage (CNG), Evry, France. RH mapping was conducted using the Carthagene software [27]. We assumed random breakage along the chromosomes and equi-probable retention of fragments. RH map was constructed in three steps: (1) a two-point analysis identified markers linked together with a LOD score greater than 8 and defined RH groups from these data; (2) using all the markers from the linkage group corresponding to GGA22, multipoint analyses were done to build a framework map using a LOD threshold of 3; and (3) additional markers were added by calculating their location relative to the framework markers. Finally the map design was created using MapChart 2.0 [28].

## Results

### Single nucleotide polymorphism genotyping

**Figure 1:**
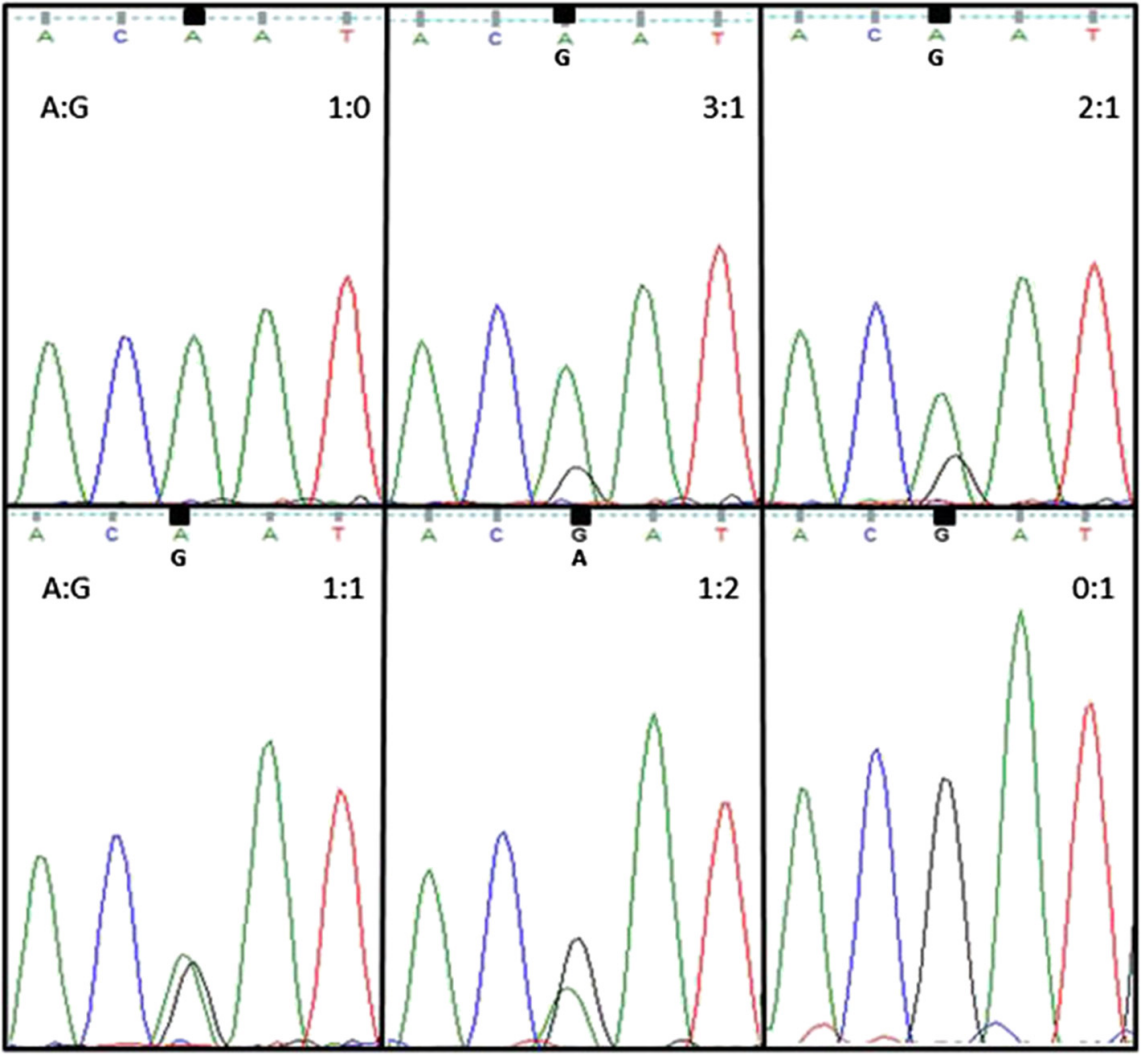
**Sequencing chromatogram of pooled DNA.** DNA from homozygous chicken (A and G allele) was mixed in 1:0, 3:1, 2:1, 1:1, 1:2 and 0:1 A to G ratios and used as PCR template and sequenced; each chromatogram peak was compared to peaks from pooled DNA samples of heritage breeds and shows the estimated A to G ratio.

Previously, we identified a SNP at nucleotide 271 of the *NK-lysin* coding sequence and discovered that the encoded protein variants have different cytotoxicities for bacteria and anticancer activity [26]. Here, we genotyped this SNP among 32 heritage breeds and 10 inbred lines. The DNA from the heritage breeds was obtained from pooled blood samples from eight to 26 animals. We compared the A and G peak heights based on the sequencing chromatogram to estimate allele frequency (Figure 1). A single A peak was detected in eight breeds and a single G peak was detected only in two of the 32 breeds (Table 2). Twenty-two of the 32 breeds analysed by pooled DNA revealed peaks for both A and G alleles. Eleven breeds showed A and G chromatogram peaks of similar height, indicating that the frequencies of the A and G alleles in the pooled DNA were approximately equal. Seven samples had a peak at least twice as high for allele A than for allele G. Only four of the 22 breeds carried G as the major allele. Thus, the A allele is more common than the G allele across all tested heritage breeds (Table 2).

**Table 2:** Heritage breeds analyzed and their genotype

**Name of breeds**	**Company**	**Nb of animals (pooled)**	**Approximate ratio of A:G**
New hamp red	Ideal Poultry	18	2:1
Silver-gray dorking	Ideal Poultry	8	1:1
Spangled russian orloff	Ideal Poultry	18	1:1
Dark brahma	Ideal Poultry	18	2:1
Standard blue cochin	Ideal Poultry	16	1:1
Astd cornish stand	Ideal Poultry	15	1:0
Dark aseel	Ideal Poultry	17	3:1
BB red malay	Ideal Poultry	16	1:1
Madagascar game	Ideal Poultry	14	4:1
Black shamos	Ideal Poultry	18	1:1
White rock	Ideal Poultry	16	2:1
Blue andalusian	Ideal Poultry	15	4:1
Gold phoenix	Ideal Poultry	15	3:1
Red shoulder yoko	Ideal Poultry	15	0:1
Crevecoeur	Ideal Poultry	15	1:0
Asst japanese bantam	Ideal Poultry	15	1:1
Rhode island red	Ideal Poultry	18	1.1
Gams modern BB red	M McMurray Hatchery	26	1:0
Sgdc silver gray dorkings	M McMurray Hatchery	17	4:1
Sums sumatras	M McMurray Hatchery	16	1:0
White faced black spanish	M McMurray Hatchery	15	0:1
Dark cornish	Cackle	15	1:2
Dominique standard	Cackle	14	2:1
Silver duckwing standard phoenix	Cackle	14	1:0
Blue cochin standard	Cackle	17	1:2
White sultan	Cackle	14	4:1
White crested black polish	Cackle	13	1:0
German spitzhauben	Cackle	15	1:0
Blue silkie bantam	Cackle	16	1:2
Silver spangled hamburg	Cackle	17	1:0
Saipan jungle fowl	Cackle	11	2:1
Egyptian fayoumis	Cackle	16	1:2

We also genotyped 10 inbred lines, eight from single animals and two from five pooled individuals. The White Leghorn line 6, Fayoumi M-5.1 and Fayoumi M-15.2 carried the G allele, while White Leghorn line 7, Leghorn Ghs-6, Leghorn Ghs-13, Spanish 21.1 and Leghorn line 8–15.1 carried the A allele. Two pooled inbred lines, line 0 and line 15Is, had the A allele (Table 3). There was no evidence of heterozygosity in any of the tested inbred lines. Thus, the A allele which has the higher antimicrobial activity was more common than the G allele across all tested heritage breeds and inbred lines.

**Table 3:** Inbred lines analyzed and their genotype

**Name of line**	**Sources**	**Number of analysed animals**	**Genotype**
Leghorn Ghs-6	Iowa State University	3	A
Leghorn Ghs-13	Iowa State University	3	A
Fayoumi M-5.1	Iowa State University and USDA-ARS Beltsville, MD	7	G
Fayoumi M-15.2	Iowa State University and USDA-ARS Beltsville, MD	7	G
Spanish 21.1	Iowa State University	3	A
Leghorn line 8–15.1	Iowa State University	3	A
Line O	USDA-ARS, East Lansing, MI	Pooled 5 individuals	A
Line15Is	USDA-ARS, East Lansing, MI	Pooled 5 individuals	A
White leghorn line 6	USDA-ARS Beltsville, MD	5	G
White leghorn line 7	USDA-ARS Beltsville, MD	5	A

### Mapping of the chicken *NK-lysin* gene

In the Gallus_gallus-4.0 Primary Assembly, the unmapped contig NW_003779909 contains the chicken *NK-lysin* along with two additional genes, *adipocyte enhancer-binding protein 1-like* (*AEBP1*) and *DNA polymerase delta subunit 2-like* (*POLD2*). We used the 6000 rad ChickRH6 panel to physically map the *NK-lysin* gene in the chicken genome. A retention frequency (RF) of 27.7% for *NK-lysin* was observed based on 25 positive PCR bands across the ChickRH6 panel. This RF value is within the range (6.8% - 55.7%) observed in other studies reported for this panel [13,29,30].

**Figure 2:**
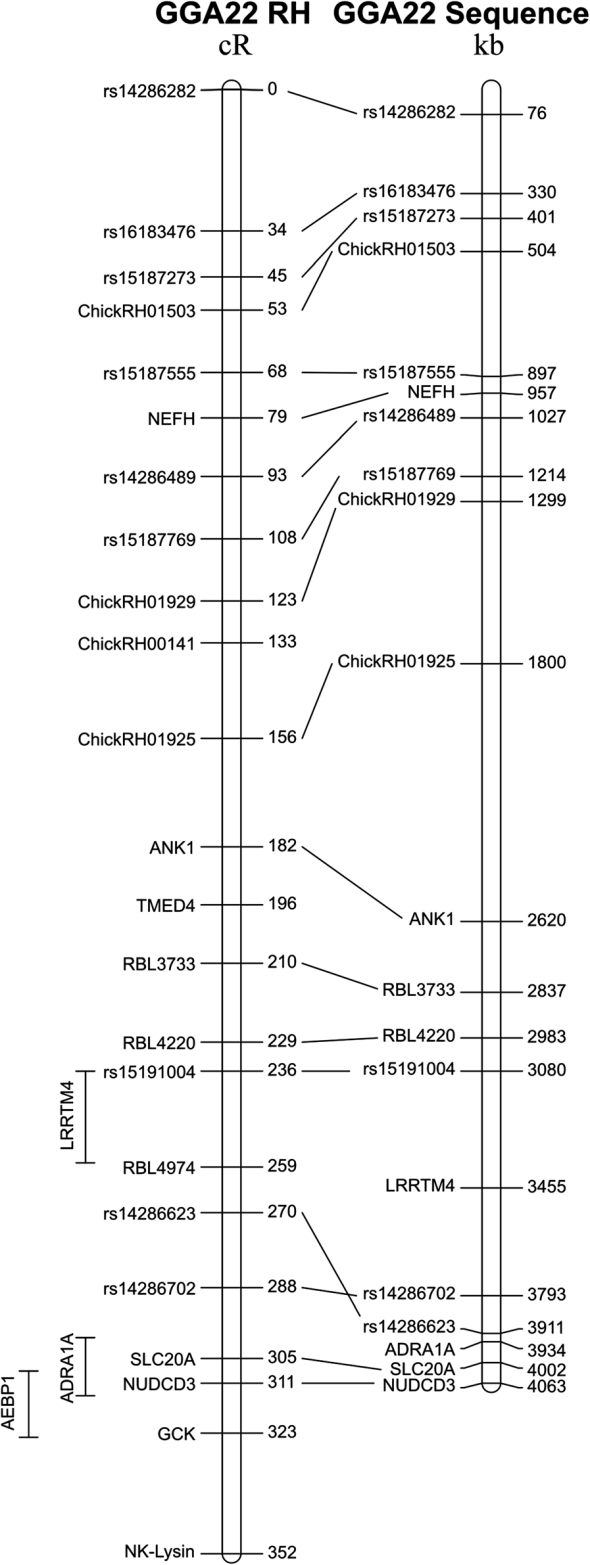
**Radiation hybrid mapping of the *NK-lysin *gene and other markers on chromosome GGA22.** The GGA22 RH map (left) is 352 cR long and is aligned to the chicken genome assembly (right); the location of the *NK-lysin* gene is indicated at the distal end of chromosome GGA22.

Using two-point analysis, the *NK-lysin* gene was included in a linkage group of 104 markers corresponding to microchromosome GGA22. After multipoint analysis, the framework map was composed of 23 markers covering 351.8 cR. Three additional markers *AEBP1*, *LRRTM4* (*leucine-rich repeat transmembrane neuronal 4*) and *ADRA1A* (*adrenoceptor alpha 1A*) were integrated at their best possible locations on the comprehensive map (Figure 2).

## Discussion

SNPs are probably the most abundant genetic variants in animal genomes and these variations can be associated with or even underlie phenotypic traits, including disease susceptibility. Previously, we identified a SNP at nucleotide 271 of the *NK-lysin* coding sequence and discovered that the encoded protein variants have different properties and also different cytotoxicities for bacteria and anticancer activity [26].

The objective of this study was to map the *NK-lysin* gene in the chicken genome and to evaluate the distribution of the SNP alleles in common chicken breeds and inbred lines. Some breeds or lines were available only as pooled DNA from multiple animals of that line. However, quantitative analysis of pooled DNA samples is recognized as a legitimate approach to approximate allele frequencies of SNPs [31]. Thus, we analyzed the peak area and height of sequencing chromatograms and compared these to a reference standard of known amounts of mixed A and G allele DNA. Since allele frequencies can only be estimated, we are unable to conclude that any breed or line is fixed for either the A or G allele. We can, however, predict very low frequencies of the minor allele in breeds for which we only detected a single peak. These analyses revealed that the A allele is more common than the G allele across all tested heritage breeds and inbred lines.

RH mapping analysis revealed that the *NK-lysin* gene is located in the distal region of chromosome GGA22. The RH map produced here is in agreement with the sequence assembly over the length of GGA22, extending it slightly with the addition of *NK-lysin*. Chromosome GGA22 is a microchromosome, approximately 4 Mb long. Microchromosomes represent about one-third of the total avian genome size, and have been found to have a higher gene density than macrochromosomes [32]. Because microchromosomes are not easy to identify cytogenetically and because they lack microsatellite markers, it is difficult to localize genes on specific microchromosomes. Thus, many of the small linkage groups in the chicken genome that have not been placed on the genetic map or genome assembly are assumed to be located on the microchromosomes [32]. RH mapping analysis revealed that the *NK-lysin* gene was located very near to the *NUDCD3* (*NudC domain containing 3*) gene on GGA22. Our data place *NK-lysin*, and the 10 kb contig NW_003779909, distal to *NUDCD3*, the most telomeric gene marker in the sequence assembly. Its location near the telomere may explain the previous difficulty in placing this contig in the assembled chicken genome sequence.

*Granulysin* (*GNLY*), the human counterpart of *NK-lysin*, is located on human chromosome 2 between the genes *SFTPB* (*surfactant*-protein B) and *ATOH8* (*atonal homolog 8*). This genomic organization is well conserved on cattle chromosome 11, pig chromosome 3, horse chromosome 15, chimpanzee chromosome 2 and dog chromosome 17. While there is no known *SFTPB* gene in the chicken genome, the *NUDCD3* gene at the telomeric end of GGA22 is located on human chromosome 7 and bovine chromosome 4. The chicken contig NW_003779909 contains the genes *NK-lysin*, *AEBP1* and *POLD2*, which are all three located on the same human chromosome 7 and bovine chromosome 4. Thus, a segment that contains these three mammalian genes is conserved in chicken, but the *NK-lysin* gene disrupts the otherwise conserved synteny.

## Conclusions

Previous reports have described the cloning of the chicken *NK-lysin* gene [24] and its antimicrobial and anticancer activity [26] but its location in the chicken genome was unknown. Here, we used ChickRH6 to localize the *NK-lysin* gene in the chicken genome at the distal end of GGA22. Two additional genes, *AEBP1* and *POLD2*, are located in the same NW_003779909 contig, and thus were also indirectly mapped to GGA22. Previously, we reported a functionally significant SNP in the *NK-lysin* coding sequence of two different chicken breeds. Here, we examined this SNP among a large number of different inbred lines and heritage breeds and found that the A allele, which has the higher antimicrobial activity, was more common than the G allele in our tested inbred lines and heritage breeds.

## Competing interests

The authors declare that they have no competing interests.

## Authors’ contributions

MOL and JEW designed the experiment and MOL, and YH performed initial RH mapping. HHC, WMM, SJL, HSL, SHL provided DNA and information on the origins of stocks and strains and contributed to writing the manuscript. MM, AV and EY constructed the refined GGA22 RH map and assigned *NK-lysin*. All authors have read and approved the final manuscript.
